# Effect of Single and Combined Expression of *Lysophosphatidic Acid Acyltransferase, Glycerol-3-Phosphate Acyltransferase*, and *Diacylglycerol Acyltransferase* on Lipid Accumulation and Composition in *Neochloris oleoabundans*

**DOI:** 10.3389/fpls.2019.01573

**Published:** 2019-11-29

**Authors:** Camilo F. Muñoz, Ruud A. Weusthuis, Sarah D’Adamo, René H. Wijffels

**Affiliations:** ^1^Bioprocess Engineering, Wageningen University and Research, Wageningen, Netherlands; ^2^Biosciences and Aquaculture, Nord University, Bodø, Norway

**Keywords:** biodiesel, triacylglycerol, *Neochloris oleoabundans*, green microalgae, glycerol-3-phosphate acyltransferase, lysophosphatidic acid acyltransferase, diacylglycerol acyltransferase

## Abstract

Microalgal lipids are promising feedstocks for food and biofuels. Since lipid production by microalgae is not yet economically feasible, genetic engineering is becoming a promising strategy to achieve higher lipid accumulation and productivities. Enzymes involved in the Kennedy pathway such as glycerol-3-phosphate acyltransferase (GPAT), lysophosphatidic acid acyltransferase (LPAT), and diacylglycerol acyltransferase (DGAT) catalyze key steps in the formation of triacylglycerol, which is the main constituent of lipids in *N. oleoabundans*. The overexpression of these enzymes in the targeted strain has a great potential to further increase their triacylglycerol content. We overexpressed single and multiple encoding genes for *LPAT*, *GPAT*, and *DGAT* from *Acutodesmus obliquus* in *N. oleoabundans*. Strains overexpressing single genes produced up to 52% and 45% g · gDW^-1^, which corresponds to 1.3- and 1.4-fold increase in total fatty acids and triacylglycerols, respectively. The orchestrated expression of the three genes resulted in 49% and 39% g · gDW^-1^, which is 1.2-folds increase in total fatty acids and triacylglycerols. Single expression of *LPAT*, *GPAT,* and *DGAT* genes resulted in higher lipid productivities during starvation without a significant effect on growth and photosynthetic activity during replete conditions. On the other hand, the simultaneous expression of *LPAT*, *GPAT,* and *DGAT* genes resulted in 52% lower growth rate, 14% lower photosynthetic activity and 4-folds increase in cell diameter. Moreover, the multigene expressing line showed a decrease in carbohydrates and protein content and an increase in pigments during nitrogen starved condition. The single and multiple expression of heterologous genes *LPAT*, *GPAT,* and *DGAT* showed to significantly enhanced the lipid accumulation in *N. oleoabundans*. Single gene expression resulted in higher lipid production and productivities without having a significant impact in the physiological status of the strains. This approach shows the potential for the generation of microalgal strains with higher economical potential for the production of lipids.

## Introduction

The continuous growth of the world population is increasing the global demand for food and fuel. Current oil production derives mainly from oleaginous crops and non-renewable fossil reserves, whose utilization causes a negative environmental impact (Alptekin et al., 2014; Moody et al., 2014; Majidian et al., 2018). It has been shown that the use of biodiesel over fossil fuels decreases greenhouse gas emissions and crop cultivation for fuel production creates a direct competition with food and feed production (Gharabaghi et al., 2015; Hanaki and Portugal-Pereira, 2018). Additionally, agricultural crop cultivation for oil production requires large amounts of arable lands and fresh-water. In the last few decades, microalgae have attracted attention for their potential to become a sustainable renewable feedstock for food and fuel production (Griffiths and Harrison, 2009; Medipally et al., 2015). Microalgae are aquatic photosynthetic microorganisms, thus capable to use sunlight and water to fix CO_2_ into biomass. Unlike higher plants, they do not need arable land and have significantly higher biomass yields and productivities (Brennan and Owende, 2010; Remmers et al., 2018). Moreover, some microalgal species can accumulate large amounts of triacylglycerol (TAG) as storage compounds, which can be converted into biodiesel by transesterification (Breuer et al., 2012; Benvenuti et al., 2017; Faried et al., 2017).

**Figure 1:**
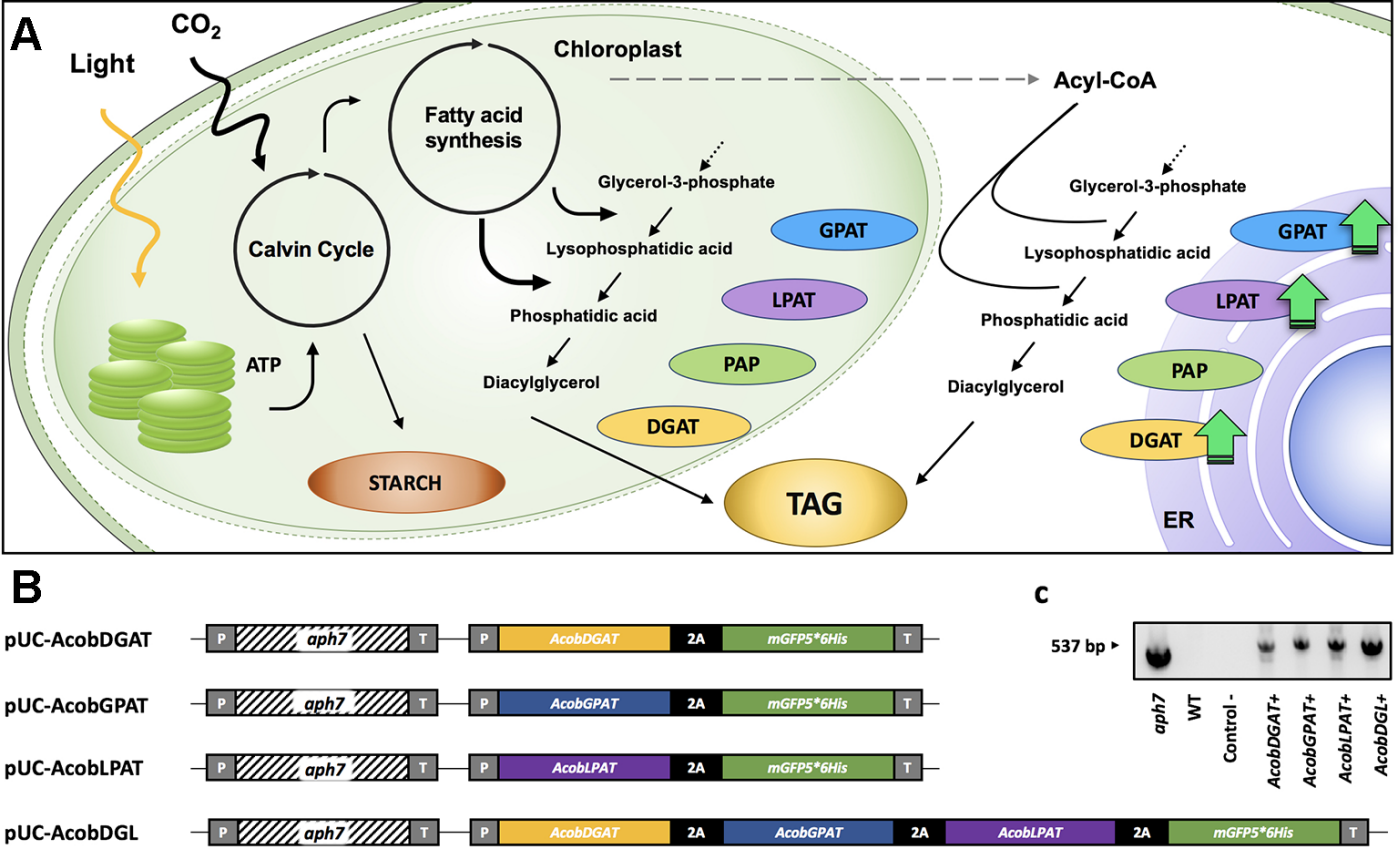
TAG biosynthesis pathway **(A)**. glycerol 3-phosphate acyltransferase (GPAT), lysophosphatidic acid acyltransferase (LPAT), phosphatidic acid phosphatase (PAP), diacylglycerol acyltransferase (DGAT). Schematic representation of expression plasmids pUC-AcobDGAT, pUC-AcobGPAT, pUC-AcobLPAT, and pUC-AcobDGL **(B)**. PCR amplification of *aph7* gene from wild type (WT) and transformant lines **(C)**. CaMV35S promoter (P), hygromycin B resistance gene *aph7*, 3´ untranslated region CaMV (T), and 2A self-cleaving peptide of foot-and-mouth disease virus (FMDV).

Oleaginous microalgae strains such as *Neochloris oleoabundans* can produce TAG up to 44% of their dry weight (Breuer et al., 2012). Since high production costs are currently involved in the process, yields, and productivities have to be improved in order to allow for full commercial scale production (Ruiz et al., 2016; Benvenuti et al., 2017). Several strategies have been proposed for their great potential to reduce the elevated costs. Efficient cultivation systems and optimization of processes and growth conditions could reduce operational costs. Additionally, costs can be reduced by selection of microalgal strains with enhanced productivities or the use of genetically improved strains which can be achieved by laboratory evolution, random mutagenesis and direct genetic manipulation (Radakovits et al., 2010; Remmers et al., 2018). Recent advances in genetic engineering and the development of new genetic tools have allowed the genetic modification of microalgae, increasing our understanding in the fatty acid biosynthesis pathways (Radakovits et al., 2010; Vazquez-Villegas et al., 2018). Several studies have shown that genes encoding key enzymes in the Kennedy pathway are potential candidates for genetic engineering to enhance TAG productivities (Banerjee et al., 2016; Ng et al., 2017). The Kennedy pathway is responsible for the synthesis of triacylglycerols in the endoplasmic reticulum (**Figure 1**). TAG synthesis starts with the acylation of glycerol-3-phosphate (G3P) by glycerol 3-phosphate acyltransferase (GPAT) to form lyso-phosphatidic acid, which is further converted into phosphatidic acid (PA) by lysophophatidic acid acyltransferase (LPAT), phosphatidic acid phosphatase (PAP), and dephosphorylates PA producing diacylglycerol (DAG). Lastly, diacylglycerol acyltransferase (DGAT) catalyzes the formation of triacylglycerols using DAG and acyl-CoA as substrates (Yu et al., 2011). The Kennedy pathway intermediates PA and DAG are also intermediate precursors for membrane lipids such as anionic phosphoglycerides (e.g. phosphatidyl serine), glycosylglycerides (e.g. galactosylglycerides), and zwitterionic phosphoglycerides (e.g. phosphatidyl choline, phosphatidylethanolamines) (Li-Beisson et al., 2019).

Previous studies have shown that the overexpression of genes encoding these enzymes resulted in increased lipid content. The overexpression of *GPAT* in *Phaeodactylum tricornutum* increased the neutral lipid content up to 42.6% of total lipids per dry weight, which corresponds to 2-folds increase in fatty acid content compared to wild type strain (Niu et al., 2016). Overexpression of *LPAT* in *Chlamydomonas reinhardtii* led to 20% increase of oil content (Yamaoka et al., 2016) and an increase of 2.3-fold (up to 13%) in TAG levels was observed when *DGAT* was overexpressed in *P. tricornutum* (Zulu et al., 2017).

In this study, we successfully expressed genes involved in the lipid biosynthesis pathway from *Acutodesmus obliquus* into *Neochloris oleoabundans*. Since *Acutodesmus obliquus* has been previously reported to accumulate up to 45% of total lipids per dry weight, we have cloned the encoding genes GPAT, LPAT and DGAT from *A. obliquus* (Breuer et al., 2013). In order to enhance lipid production in *N. oleoabundans,* we performed the single and combined gene expression of the three genes. In addition, we further investigated and characterized growth, lipid composition, and cellular biochemical composition of all transformant lines obtained.

## Materials and Methods

### Microalga Strain and Culture Conditions

*Neochloris oleoabundans* UTEX 1185 was obtained from the University of Texas Culture Collection of Algae (UTEX). *N. oleoabundans* strain was grown in Freshwater (FW) medium as described by Breuer et al. (2012). Prior to experiments, cultures were maintained in shake flasks at 25°C on light:dark cycles of 16:8 h under a light intensity of 40 µmol m^-2^ s^-1^ on a rotary shaker (125 rpm). During nitrogen starvation experiments, *N. oleoabundans* was grown in 500 ml Erlenmeyer flasks. Cultures were placed in a shaker incubator operating at 25°C with light intensities of 150 µmol m^-2^ s^-1^ (light:dark cycles of 16:8), enriched with 5% CO_2_, and continuous agitation of 150 rpm.

*Escherichia coli* NEB-Alpha5 (New England Biolabs) was grown at 37°C in Luria-Bertani (LB) broth or agar supplemented with 100 mg · L^-1^ of ampicillin as needed.

### Plasmid Construction

The plasmids pUC-AcobLPAT, pUC-AcobGPAT, and pUC-AcobDGAT were constructed for the single expression of *LPAT*, *GPAT,* and *DGAT*. In order to overexpress all three genes simultaneously we constructed the expression vector pUC-AcobDGL. The plasmid pUC19 was obtained from Addgene and used as a backbone in all the constructs. pUC19 was digested with HindIII and NdeI restriction enzymes (Thermo Scientific) in order to linearize and remove the multiple cloning site of the vector. The cassettes CaMV35Sp-Aph7-Ter and CaMV35Sp-2A-GFP-Ter were synthetized and assembled into the linearized pUC19. Both cassettes contain the cauliflower mosaic virus (CaMV) 35S promoter and terminator. Moreover, the cassette CaMV35Sp-2A-GFP-Ter contains a NedI restriction site between the promoter and the 2A self-cleaving sequence for insertion of genes of interest. The plasmid assembly was performed using HiFi DNA Assembly Master Mix (NEB) according to the manufacturer’s instructions and transformed into NEB-Alpha5 *E. coli* competent cells (New Englands Biolabs). The construct containing both cassettes (pUC19-aph7-2A-GFP) was digested by NdeI restriction enzyme. Synthetized *LPAT*, *GPAT,* and *DGAT* containing overlap regions with pUC19-aph7-2A-GFP were inserted *via* HiFi assembly to generate pUC-AcobLPAT, pUC-AcobGPAT, and pUC-AcobDGAT. In order to generate the plasmid pUC-AcobDGL, we synthetized the sequences DGAT-2A, GPAT-2A, and LPAT. All synthesized sequences contained overlap regions and stop codons of each gene were replaced by 2A self-cleaving peptide of foot-and-mouth disease virus (FMDV). The three genes were assembled into linearized pUC19-aph7-2A-GFP by Hifi assembly and transformed into NEB-Alpha5 *E. coli* competent cells (New Englands Biolabs). The sequences encoding for LPAT, GPAT, and DGAT were obtained from the draft genome of *Acutodesmus obliquus* (Carreres et al., 2017) by sequence analysis (NCBI database) using Basic Local Alignment Search Tool (BLAST). All the sequences were identified as putative and no specific intracellular localization was predicted when using the prediction tool PredAlgo (Tardif et al., 2012).

### Transformation of *N. oleoabundans* by Electroporation

*N. oleoabundans* cells were harvested and washed in electroporation buffer as described by Muñoz et al. (2018). The transformation mixture containing 2 µg · ml^-1^ of linearized plasmid and 25 µg · ml^-1^ of boiled salmon sperm DNA (D1626, Sigma) were incubated on ice for 15 min. The electroporation was performed in 2-mm electroporation cuvettes by applying 6 kV · cm^-1^. Electroporated cells were recovered in the dark, transferred into 10 ml of FW medium and incubated overnight on dark at 25°C on a rotary shaker (125 rpm). After recovery, cells were harvested, re-suspended in 200 µl of FW medium and plated onto FW agar plates containing 50 µg · ml^-1^ of hygromycin B. Plates were incubated at 25°C, under light intensities of 60 µmol · m^-2^ s^-1^ (light:dark cycle cycles of 16:8) and supplemented with 2.5% CO_2_.

### Selection and Screening of Transformant Lines

Selection of transformant lines was performed on FW agar plates containing 50 µg · ml^-1^ of hygromycin B. Antibiotic resistant colonies obtained on plate were transferred to new plates containing 75 µg · ml^-1^ of hygromycin B. Identification of positive transformants was performed by PCR amplification of the hygromycin resistance gene (*aph7*) with primers used by Munoz et al. (2018). DNA extraction and colony PCR were performed by using Phire Plant Direct PCR Master Mix (ThermoFisher Scientific). Colonies obtained on selective plates were transferred to 20 µl of dilution buffer (provided in the kit), mixture was vortexed for 30 s and 0.5 µl was used as DNA template in the PCR reaction. Phire hot start II DNA polymerase was used for the PCR amplification of *aph7* gene following the manufacturer’s instructions. Positive transformants were inoculated in liquid FW medium containing 75 µg · ml^-1^ of hygromycin B and grown in 48 microwell plates. Fast growing transformants were identified by optical density measurements at 750 nm. Moreover, we visualized transformants by fluorescence microscopy and selected transformants with high green fluorescence signals for subsequent analysis. Detection of green fluorescent protein was performed with the fluorescence microscope EVOS FL Auto Cell Imaging System incorporating a GFP excitation/emission cube (EVOS FL, ThermoFisher Scientific).

### Growth, Cell Diameter, Cell Number, and Dry Weight Determination

The optical density was monitored at a wavelength of 750 nm using a UV-VIS spectrophotometer (Hach Lange DR6000). Cell concentration and cell size were measured in triplicate with the Beckman Coulter Multisizer III (Beckman Coulter Inc., USA) using a 50 µm aperture tube. Microscopic analysis was performed with the microscope EVOS FL Auto Cell Imaging System (EVOS FL, ThermoFisher Scientific). All samples were diluted 200 times in Isotone^®^ II diluent solution before measurements. Dry weight concentration was determined by filtrating 10 ml of culture broth on a pre-dried and pre-weight 55 mm Whatman glass fibre filter paper (GF/F; Whatman International Ltd, Maidstone, UK). The filter was washed with filtered demineralized water and subsequently dried overnight at 100 °C before weighing. The dry weight of the samples was calculated from the difference in weight between the dry filters with and without biomass.

### Measurement of Photosynthetic Activity

Quantum yield (Fv/Fm) was measured by chlorophyll a fluorescence at 455 nm using a fluorometer AquaPen-C AP-C100 (Photon Systems Instrument, Czech Republic). Samples were diluted to an OD_750_ of 0.5 and adapted to dark for 15 min at room temperature before measurement (Janssen et al., 2018).

### Determination of Lipid Content and Fatty Acid Composition

Total fatty acid content and lipid composition were determined as described by Breuer et al. (2013). Lipids were extracted from 10 mg of freeze dried biomass. Cells were mixed with chloroform:methanol (1:1.25, v:v) containing tripentadecanoin (T4257, Sigma Aldrich) and 1,2−dipentadecanoyl−sn−glycero−3−phospho−(1’−rac-glycerol) (sodium salt) (840434, Avanti Polar Lipids Inc.) as internal standard for TAG and polar lipid fraction, respectively. Biomass was disrupted using a beat beater, chloroform:methanol was subsequently evaporated and the extract was dissolved in hexane:diethylether (7:1 v/v). Neutral or apolar lipids containing triacylglycerols were separated from polar lipids using a Sep-Pak Vac silica cartridge (6cc, 1g, Waters). Apolar lipids were extracted by loading 10 ml hexane:diethylether (7:1 v/v) and polar lipids by loading 10 ml of methanol:acetone:hexane (2:2:1 v/v/v). Both lipid extracts were quantified using a gas chromatography (GC-FID) as described by Breuer et al. (2013). The total apolar and polar lipid content was calculated as a sum of the individual fatty acids of these fractions.

### Carbohydrates Determination

The total carbohydrate content was determined according to DuBois et al. (1956) and Herbert et al. (1971). Phenol-sulphuric acid was added to 10 mg of freeze dried microalgae and absorbance was measured at 483 nm. Glucose monohydrate was used as a standard.

### Protein Determination

The total protein content was determined using a colorimetric assay (Bio-Rad DC protein assay) according to manufacturer’s instructions. Total protein content was analysed in 10 mg of freeze dried biomass and bovine serum albumin (BSA) was used as a standard.

### Pigment Determination

Pigment composition was determined as described by Lichtenthaler (1987). The experiment was performed in triplicate and absorbance was measured at 470, 652, and 665 nm. Chlorophyll a, chlorophyll b, total chlorophyll, and total carotenoids were calculated using Arnon’s equations.

### Calculations

The average and maximum volumetric biomass productivities were calculated as described by Breuer et al. (2012). The growth rate was calculated using the equation *µ* = ln(X_2_/X_1_)/(*t*_2_ − *t*_1_), where *µ* is the specific growth rate, X_1_ and X_2_ are the biomass (dry weight) at time 1 (*t*_1_) and time 2 (*t*_2_), respectively.

### Statistical Analysis

All the experiments in this study were performed in biological triplicates. The data were represented as mean ± standard deviation (SD). Statistical analysis were performed using the student’s *t* test and a *P* value < 0.05 was considered statistically significant.

## Results

### Experimental Design, Plasmid Construction and Selection of Transformants

In order to increase triacylglycerol content in *N. oleoabundans*, we constructed four expression vectors. The plasmids pUC-AcobDGAT, pUC-AcobGPAT, and pUC-AcobLPAT were constructed for the single expression of the genes encoding DGAT, GPAT, and LPAT, respectively. The plasmid pUC-AcobDGL was constructed in order to assess the simultaneous expression of the three genes (**Figure 1**). The expression of selectable marker (*Streptomyces hygroscopicus* aminoglycoside phosphotransferase gene *aph7*, inferring resistance to hygromycin), reporter gene (encoding green fluorescence protein, mGFP5), and genes of interest were regulated under the CaMV35S promoter and terminator. Moreover, a 2A self-cleaving peptide of foot-and-mouth disease virus (FMDV) was used for polycistronic expression of the gene(s) of interest and mGFP5, in both the single and multiple gene expression vectors. In general, viral‐derived 2A peptides are used for enabling a single transcript to translate discrete protein products of multiple transgenes in eukaryotes and are widely used to tie resistance markers to the production of target proteins. Having a fluorescent protein (mGFP5) at the end of the 2A polycistronic construct confers the capability to directly select for positive transformant lines. The selection for positive GFP fluorescence shows clear evidence of concomitantly expression of all the upstream genes (Daniels et al., 2014; Wang et al., 2015; Liu et al., 2017; Poliner et al., 2018). Around 200 hygromycin B resistant colonies were obtained on plates containing FW agar medium and 50 µg · ml^-1^ hygromycin B. Only 15% of the transformant lines survived when transferred to a higher antibiotic concentration, which were confirmed to contain the hygromycin B resistance gene *aph7* (**Figure 1C**). We then identified the fast-growing transformants with high green fluorescence signals on liquid medium by measuring OD_750_ and using a fluorescence microscope, respectively, and we selected them for subsequent analysis. The selected positive transformants expressing the single genes *DGAT*, *GPAT,* and *LPAT* from *A. obliquus* were named AcobDGAT+, AcobGPAT+, and AcobLPAT+, respectively. The name AcobDGL+ was assigned to the *N. oleoabundans* cell lines expressing the three genes simultaneously.

### Growth Analysis and Characterization of Engineered Strains

**Figure 2:**
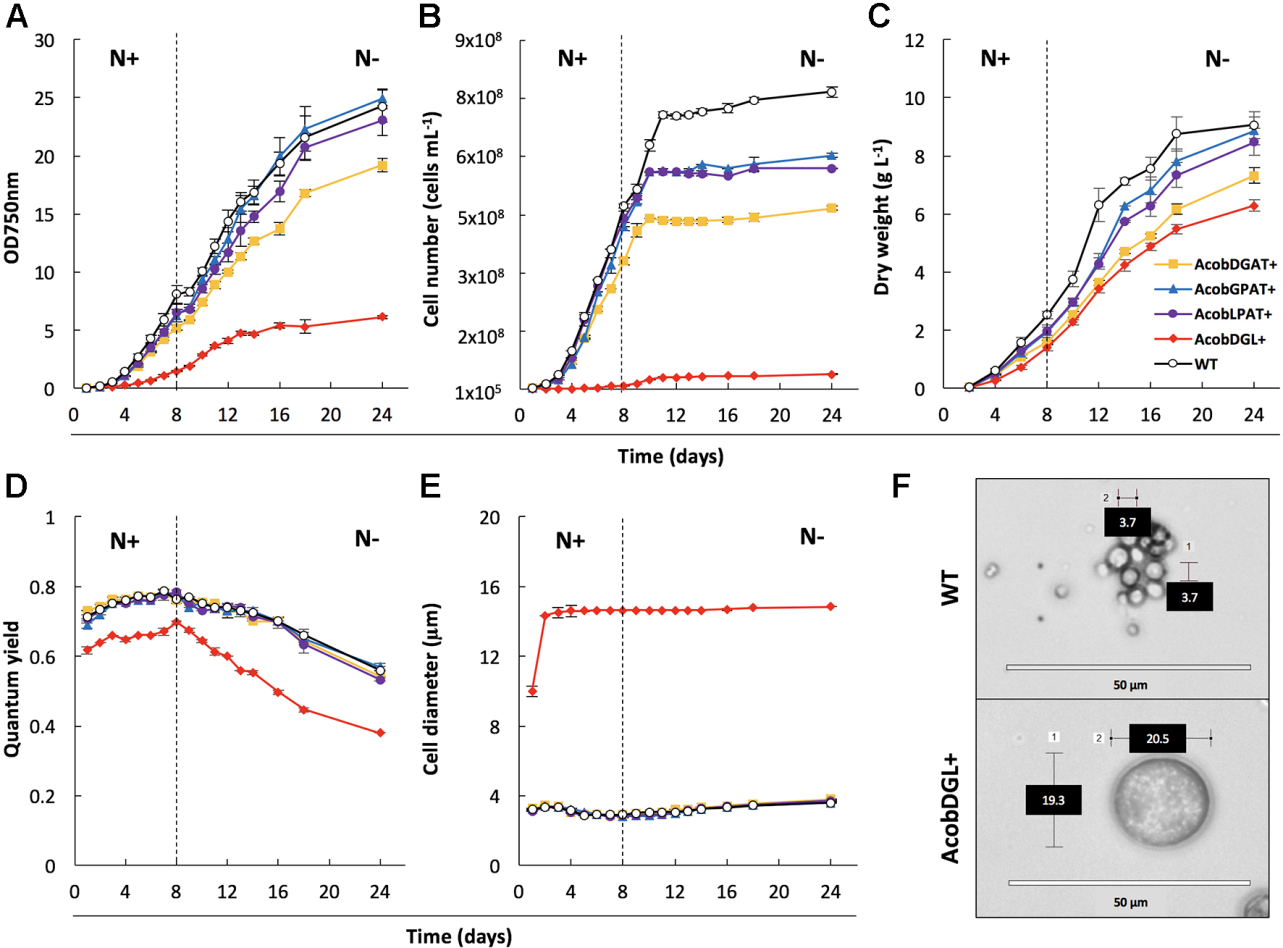
Analysis of cell growth and photosynthetic efficiency. Measurements of optical density at 750 nm **(A)**, cell number **(B)**, dry weight **(C)**, quantum yield **(D)** and cell diameter **(E)** in wild type (WT) strain and transformant lines. Microscopic image of wild type strain and *N. oleoabundans* expressing *DGAT*, *GPAT,* and *LPAT* simultaneously **(F)**. Dashed lines indicate the moment at which nitrogen starvation started. The data represent the average of n = 3 replicate experiments. Standard deviation bars are shown.

In order to characterize and determine the effect of gene overexpression in the selected transformant lines, we analyzed growth, morphology, and photosynthetic efficiency (**Figure 2**). Biological replicates were cultivated in flasks under standard growth conditions and then transferred to nitrogen-depleted medium. During replete and deplete conditions all transformant lines showed lower cell numbers per volume compared to the wild type strain (**Figure 2B**). Although the cell numbers were lower, AcobGPAT+ and AcobLPAT+ showed similar optical densities and dry weights compared to the wild type during nitrogen replete and deplete conditions (**Figures 2A–C**). On the other hand, the strains AcobDGAT+ and AcobDGL+ showed significantly lower values of optical density, cell number, and dry weight (end-point *P* value < 0.05).

To determine the effect of gene overexpression on photosynthetic performance, we monitored the chlorophyll fluorescence Fv/Fm of transformant lines and compared them with the *N. oleoabundans* wild type line. Compared to the control, no significant differences were observed in photosynthetic efficiency in the lines overexpressing single genes (end-point *P* value < 0.05). Wild type, AcobDGAT+, AcobGPAT+, and AcobLPAT+ showed an increase in quantum yield from 0.7 to 0.8 when grown in replete conditions, which decreased in deplete conditions to a minimum value of 0.6 at the end point of measurement. However, the three-gene overexpressing line AcobDGL+ showed a significantly lower quantum yield value (end-point *P* value <0.05), reaching the lowest values of 0.6 during replete conditions and 0.4 during nitrogen deplete conditions (**Figure 2D**). We further investigated the cell diameter of all transformant and wild type lines by microscopic analysis. Control and transformant lines overexpressing single genes showed no differences in cell size and during replete and deplete conditions they had cell diameters between 3.7 and 4.0 µm. On the contrary, AcobDGL+ was considerably larger (**Figure 2E**), showing an average size around 14 µm in cell diameter, with some cells reaching 20 µm in diameter (**Figure 2F**).

### Biochemical Composition of Engineered Strains

Carbohydrates, proteins, and pigments were analyzed in transformant and wild type control lines during cultivation in standard and nitrogen-starved conditions.

**Figure 3:**
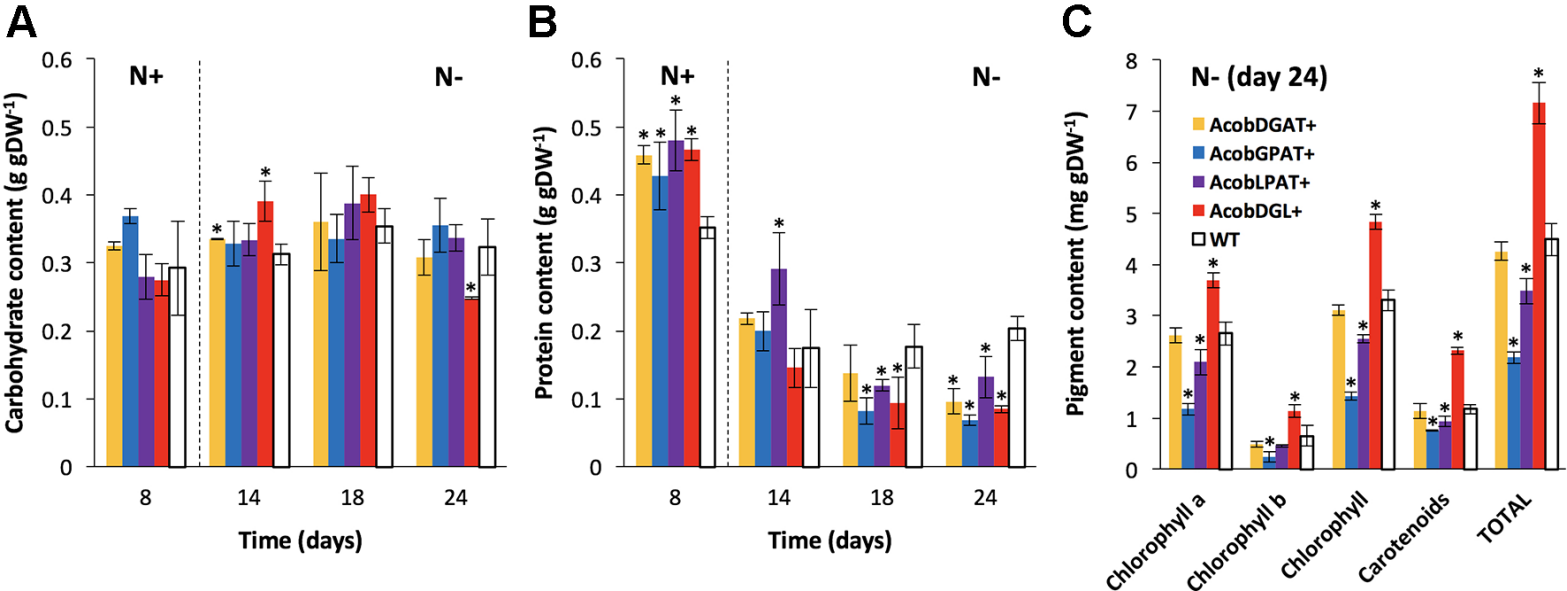
Biochemical composition in *N. oleoabundans* wild type strain and transformant lines. Carbohydrate **(A)**, protein **(B)**, and pigment **(C)** content in wild type (WT) and transformant lines. Dashed lines indicate the moment at which nitrogen starvation started. Pigment content as determined for all strains at day 24 under nitrogen starvation. Chlorophyll represents the sum of chlorophyll a and chlorophyll b. Total pigments correspond to the sum of total chlorophyll and carotenoids. The data represent the average of n = 3 replicate experiments. Standard deviation bars are shown. Significant difference between wild type strain and transformant lines is indicated at *P* < 0.05 (*).

As shown in **Figure 3A**, no substantial differences were observed in carbohydrate content between the wild type and transformant lines during replete conditions. During deplete conditions, AcobDGL+ showed the lowest carbohydrate content of 0.24 g · gDW^-1^ at day 24, while no differences were observed in the lines expressing single genes and control.

In the wild type control line, a maximum of 0.35 g · gDW^-1^ and a minimum of 0.20 g · gDW^-1^ of protein content was reached during replete and deplete conditions, respectively (**Figure 3B**). Interestingly, the total protein content was higher in all transformant lines during nitrogen replete conditions and lower during starvation, compared to wild type. Furthermore, we analyzed chlorophyll a, chlorophyll b, and carotenoid content at day 24 during nitrogen deplete conditions. As shown in **Figure 3C**, AcobDGAT+ showed similar chlorophyll and carotenoid content (total pigment content of 4.5 mg · gDW^-1^) compared to wild type. AcobGPAT+ and AcobLPAT+ resulted in lower pigment content, being AcobLPAT+ the lowest with 2 mg · gDW^-1^. Interestingly, AcobDGL+ showed higher values in chlorophyll and carotenoid content reaching 7.5 mg · gDW^-1^ of total pigment content compared to the control.

### Effect of Gene Expression on Fatty Acid Synthesis

**Figure 4:**
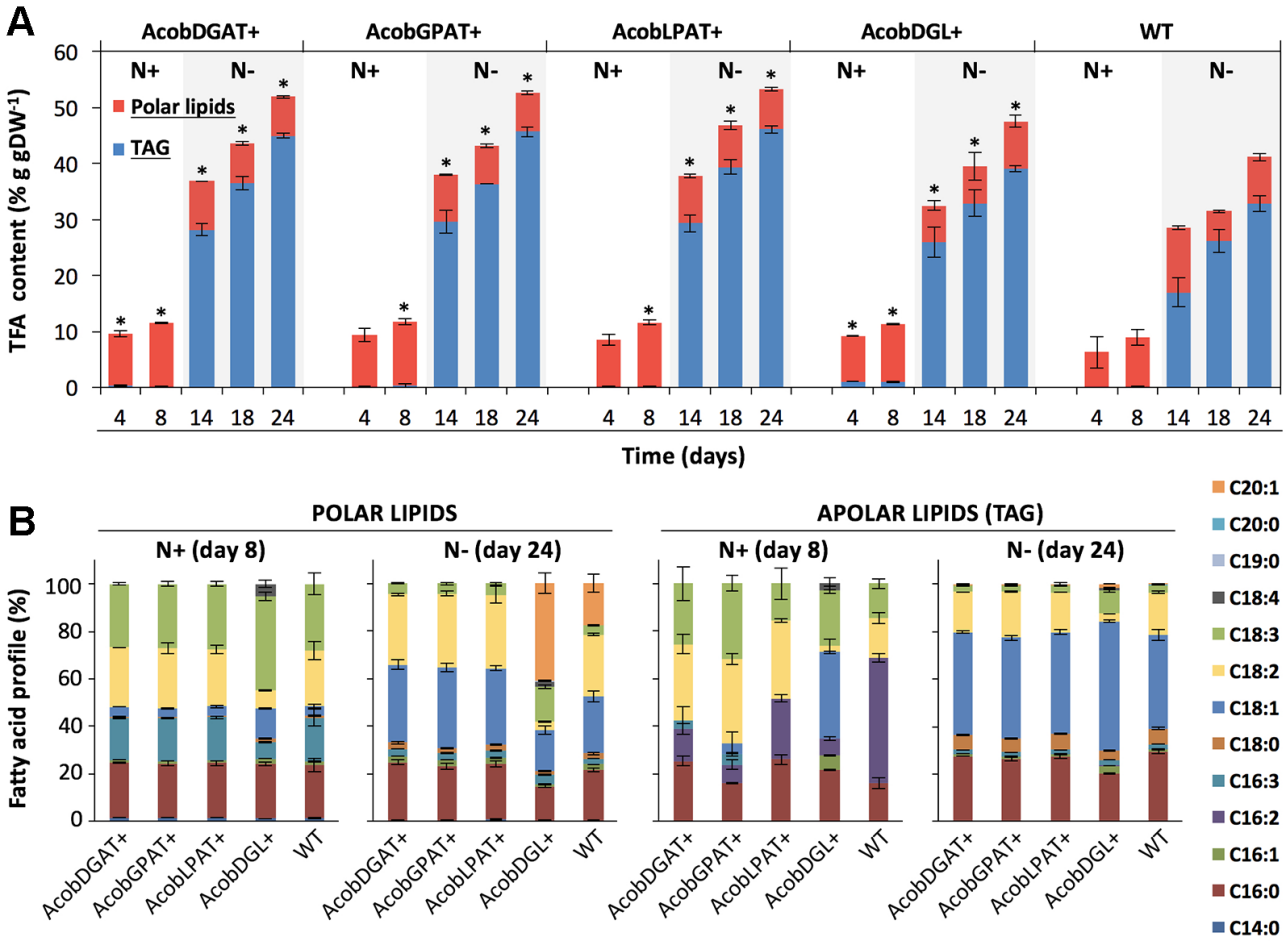
Total fatty acid (TFA), triacylglycerols (TAG), and fatty acid composition in *N. oleoabundans* wild type strain and transformant lines. Total fatty acids: TAG and polar lipids **(A)**. Polar and apolar lipid (TAG) composition at days 8 and 24 during nitrogen replete (N+) and depleted (N-) nitrogen conditions, respectively **(B)**. The data represent the average of n = 3 replicate experiments. Standard deviation bars are shown. Significant difference between wild type strain and transformant lines is indicated at *P* < 0.05 (*).

The total fatty acid (TFA) and triacylglycerol (TAG) contents were analyzed throughout nitrogen replete and deplete conditions. As shown in **Figure 4A**, the total fatty acid content during nitrogen replete conditions was similar among transformant and wild type control lines. Only AcobDGL+ showed an increase in TAG content, having 0.9% g · gDW^-1^ of TAG compared to 0.1% g · gDW^-1^ present in AcobDGAT+, AcobGPAT+, AcobLPAT+, and wild type. The maximum TFA and TAG content was achieved at day 24 during nitrogen deplete conditions for all the transformant lines and wild type. In these conditions, the expression of the single genes encoding DGAT, GPAT, and LPAT showed the highest TFA and TAG content at around 52% and 45% g · gDW^-1^, respectively. The simultaneous overexpression of the three genes in AcobDGL+ showed lower TFA and TAG (49% and 39% g · gDW^-1^ respectively) compared to the single gene expression, but higher compared to the wild type control line. We also analyzed the total fatty acids composition of the polar and apolar (TAG) fraction (**Figure 4B**). Although the total polar lipid content of the transformant lines during replete and deplete conditions was similar to wild type, it showed differences in composition. In particular, during nitrogen replete conditions, we observed an increase in C18:3 and decrease in C16:3 and C18:2 fatty acids in the polar lipid fraction for AcobDGL+, and no significant difference for the single gene expressing lines compared to wild type. During nitrogen deplete conditions, interestingly, no C20:1 was produced in AcobDGAT+, AcobGPAT+, and AcobLPAT+, while they showed an increase of C18:1 compared to wild type. Differently, AcobDGL+ showed 2.4 and 3.9-fold increase in C20:1 and C18:3 fatty acids content, respectively, with a reduced C16:0, C18:1, and C18:2 content, compared to wild type.

Similarly, also the apolar (TAG) fraction presented differences in composition. During replete conditions, AcobDGL+ showed an increase in C18:1 and the presence of C18:4 and C16:1. For the strains expressing single genes we observed a substantial decrease in C16:2 and increase in C18:2 in the apolar (TAG) fraction. Moreover, AcobDGAT+, AcobGPAT+, and AcobDGL+ showed the presence of 16:3, and AcobDGAT+, AcobLPAT+, and AcobDGL+ had higher C16:0 content compared to wild type. Under deplete conditions, the apolar lipid composition of the transformant lines expressing single genes showed a similar distribution compared to wild type, while AcobDGL+ showed an increase in C18:3, C18:1, and C20:1 fatty acids and a decrease in C16:0 and C18:2 fatty acids.

### Impact of Single and Multi-Gene Expression on Biomass and Fatty Acid Productivities

In order to determine performance of all the tested lines, we calculated the volumetric biomass, total fatty acid, and triacylglycerol productivities. As shown in **Table 1**, during nitrogen replete conditions, *N. oleoabundans* wild type showed high volumetric biomass productivity (413 mg · L^-1^ d^-1^) and growth rate (1.23 d^-1^). The most affected in both volumetric biomass productivity (231 mg · L^-1^ d^-1^) and growth rate (0.59 d^-1^) was the three-gene expressing line AcobDGL+. Under nitrogen-depleted conditions, AcobDGAT+, AcobGPAT+, AcobLPAT+, and wild type reached higher volumetric biomass productivities compared to AcobDGL+. The highest volumetric TFA and TAG productivities were obtained during nitrogen-depleted conditions for all the lines. However, remarkably, AcobGPAT+ and AcobDGL+ showed higher volumetric TAG productivity in replete conditions, which were 1- and 4.5-fold higher than wild type, respectively. Under deplete conditions, all the lines expressing single genes showed high TFA and TAG productivities compared to AcobDGL+ and wild type, with AcobLPAT+ reaching the highest values (248.0 and 235 mg· L^-1^ d^-1^, respectively).

**Table 1:** Growth rate and average volumetric productivities of biomass, total fatty acids (TFA), and triacylglycerols (TAG) for nitrogen replete (N+) and nitrogen deplete (N-) conditions.

Strains	Volumetric biomass productivity (mg · L^-1^ d^-1^)	Volumetric TFA productivity <br/>(mg · L^-1^ d^-1^)	Volumetric TAG productivity<br/> (mg · L^-1^ d^-1^)	Growth rate (d^-1^)
WT N+	413.1 ± 50	46.7 ± 8	0.6 ± 0.1	1.23 ± 0.01
WT N-	386.5 ± 35	172.6 ± 4	192.4 ± 21	–
AcobDGAT+ N+	254.1 ± 12	34.9 ± 6	0.4 ± 0.1	1.01 ± 0.01
AcobDGAT+ N-	383.1 ± 26	212.9 ± 31	202.5 ± 38	–
AcobGPAT+ N+	321.7 ± 25	46.1 ± 8	1.3 ± 0.2	1.04 ± 0.01
AcobGPAT+ N-	422.2 ± 44	230.2 ± 20	224.2 ± 32	–
AcobLPAT+ N+	324.4 ± 36	46.4 ± 8	0.5 ± 0.1	1.05 ± 0.01
AcobLPAT+ N-	426.5 ± 40	248.0 ± 26	235.3 ± 42	–
AcobDGL+ N+	231.8 ± 23	33.5 ± 6	2.7 ± 0.5	0.59 ± 0.01
AcobDGL+ N-	307.9 ± 33	167.0 ± 42	142.5 ± 47	–

The data represent the average of n = 3 replicate experiments.

## Discussion

The oleaginous microalga *Neochloris oleoabundans* has been previously shown to be an industrially relevant strain for biofuel production due to its ability to produce large amounts of lipids, mainly in the form of TAGs, during stress conditions (i.e. nitrogen deprivation) (Li et al., 2008; de Jaeger et al., 2018). With the recent advances in biotechnology, tools and techniques for the genetic modification of microalgae are expanding quickly and they could be used for the generation of improved strains with enhanced lipid productivities. It has been demonstrated that overexpression of genes involved in the Kennedy pathway in microalgae species such as *Chlamydomonas reinhardtii*, *Phaeodactylum tricornutum,* and *Nannochloropsis oceanica* can lead to higher lipids content. Overexpression of the endogenous LPAT encoding gene in *C. reinhardtii* resulted in an increase of 20% in total fatty acids (Yamaoka et al., 2016). Similarly, the overexpression of endogenous GPAT encoding gene in *P. tricornutum* led to an enhanced neutral lipid content (2-fold higher than wild type) and specifically, to a significant increase in unsaturated fatty acid profile (Niu et al., 2016). Moreover, the DGAT enzyme, responsible for the last step in TAG biosynthesis, has been regarded as a promising target for improving TAG content in multiple studies. Deng et al. (2012), previously showed that overexpression of the endogenous DGAT encoding gene in *C. reinhardtii* can result in an increase of total fatty acids by 48% and 43% of its dry weight when grown under nitrogen replete and deplete conditions, respectively.

In our study, we report the expression of LPAT, GPAT, and DGAT encoding genes from the microalga *Acutodesmus obliquus* in *N. oleoabundans*. Our results demonstrate that the single expression of LPAT, GPAT, and DGAT encoding genes lead to higher lipid accumulation per dry weight during deplete conditions compared to wild type, mainly due to a ∼1.3-fold increase in their TAG content. The lipid composition of the apolar fraction remains unchanged compared to wild type, being oleic acid (C18:1), palmitic acid (C16:0), and linoleic acid (C18:2) as the main constituents. Although single gene expression did not lead to higher lipid content during nitrogen replete conditions, we observed a different fatty acid distribution in the apolar fraction. In particular, hexadecadienoic acid (C16:2) decreased and linoleic acid (C18:2) increased substantially compared to wild type. The single expression of *LPAT*, *GPAT,* and *DGAT* genes showed no changes in polar lipid distribution during nitrogen replete conditions. However, during nitrogen depleted conditions all the three transformant lines seem to favor the production of oleic acid (C18:1) at the expenses of eicosenoic acid (C20:1), which diminished, compared to wild type.

In our attempt to increase the lipid content in *N. oleoabundans* even further, we overexpressed the three LPAT, GPAT, and DGAT encoding genes simultaneously. Unexpectedly, in this case we saw an increase in the total fatty acids content of a factor of 1.2-folds compared to wild type, which was lower compared to what we have observed for the single expressing lines. Also in this case, the major increase was observed in the TAG fraction (**Figure 4A**). In particular, the apolar fraction (TAG) of the multiple gene expressing line contained an increase in C18:1 and C18:3 fatty acids, and a reduction in C18:0, C16:0 and C18:2, compared to the single expressing lines and wild type apolar lipid distribution. This suggests that the orchestrated expression of these enzymes shifts the lipid composition towards long unsaturated fatty acid chains. Oleic acid (C18:1) is the major fatty acid produced by *N. oleoabudans* and *A. obliquus*. Moreover, in *A. obliquus* fatty acid profile linolenic acid (C18:3) has higher relative abundance compared to C18:2 (Breuer et al., 2013). Taken together, these findings may suggest a preference of *A. obliquus* enzymes for both C18:1 and C18:3 fatty acids to be integrated in the triacylglycerols of *N. oleoabundans*. It also suggests that these enzymes are interfering with other lipid metabolic pathway, such as fatty acid elongation and desaturation. Furthermore, also during nitrogen replete conditions the apolar fraction showed an increase in oleic and linolenic fatty acids. Interestingly, the simultaneous gene expression led to substantial changes in the fatty acid profile of the polar fraction. Since the main component of cellular membranes are polar lipids, we hypothesize that the alteration in the lipid distribution might also be correlated to the significant increase in cell size of the multiple gene expressing line. The Kennedy pathway enzymes share precursor like PA and DAG with the enzymes for the biosynthethic pathway of the membrane lipids, such as phosphoglycerides and glycosylglycerides. Further investigations could be focused in understanding which lipid species and how the intermediate precursor pools are affected by the enhanced expression of a heterologous Kennedy pathway, which could elucidate the lipid metabolism in microalgae.

In order to determine whether overexpression of *LPAT*, *GPAT,* and *DGAT* genes had an effect on biomass and fatty acid productivities we calculated the volumetric productivities of all transformants. The highest volumetric TFA and TAG productivities were obtained during nitrogen-starved conditions by single gene expressing lines. Moreover, the expression of single genes led to the highest TFA and TAG productivities, compared to the multiple gene expression and wild type. Previous studies reported that the heterologous expression of *DGAT* in *Scenedesmus obliquus* and *Phaeodactylum tricornutum* increased lipid content and lipid productivities (Chen et al., 2016; Zulu et al., 2017). The dual overexpression of the endogenous *LPAT* and *GPAT* in *P. tricornutum* and the multiple expression of the heterologous *LPAT*, *GPAT*, *PAP,* and *DGAT* in *Chlorella minutissima* showed an enhanced lipid content and productivities as well (Hsieh et al., 2012; Wang et al., 2018). Previous studies have reported no effect on growth when overexpressing genes involved in the Kennedy pathway. In our study, the highest volumetric biomass productivity and highest growth rate were achieved by *N. oleoabundans* wild type during nitrogen replete conditions. During nitrogen deplete conditions the specific growth rate was similar among the single overexpressing lines and wild type, while the transformant line expressing the three genes simultaneously was the most affected, with a growth rate reduced by half, compared to the replete conditions. Conversely, Wang et al. (2018) recently reported that the dual expression of *GPAT* and *LPAT* in *P. tricornutum* enhanced growth rate during the mid-log phase, however this was not observed in our transformant line (**Figure 2B**).

In previous reports, it has been shown that the overexpression of these single enzymes did not affect the photosynthetic efficiency of the transformant lines. Thus, it has been hypothesized that the increased availability of fatty acid precursors for TAG synthesis, such as PA, induced by the overexpression of these enzymes, may also serve as precursors for the synthesis of plastidic membranes, resulting in elevated photosynthetic efficiencies (Niu et al., 2013; Niu et al., 2016; Balamurugan et al., 2017). In our study, we did not observe a significant change in photosynthetic efficiencies in the lines overexpressing single genes, compared to wild type. On the other hand, the multiple gene expression showed a significantly reduced photosynthetic activity, indicating that the introduction of these three genes results in an increased metabolic load that has a direct impact on the physiological status of the cells. In this regard, the reduced quantum yield and size enlargement in this transformant line may also be related to a stress component, which could be correlated to the alteration of both polar and apolar lipid compositions, interestingly different compared to wild type and the single gene expressing lines.

We further investigated the biochemical composition of all transformant lines in order to determine the effect of gene expression on the synthesis of cellular components. Our results showed that only when the three genes were overexpressed the carbohydrate content was reduced at the end of the experiment. Moreover, all the transformant lines showed a reduced protein content during nitrogen depleted conditions. Similarly, Balamurugan et al. (2017) and Wang et al. (2018) reported that overexpression of *GPAT* or dual expression of *GPAT* and *LPAT* in *P. tricornutum*, respectively, led to lower protein and carbohydrate content in starved conditions. These results suggest that carbohydrate and protein metabolism are redirected towards lipid production, and in particular, the orchestrated expression of the three genes has a stronger impact in redirecting the carbon flux from carbohydrates to lipids. On the other hand, during nitrogen replete experiments, we observed higher protein content compared to the wild type strain as a result of the high expression of *LPAT*, *GPAT,* and *DGAT* genes, which is in accordance with previous studies (Dinamarca et al., 2017). Other cellular components such as chlorophylls and carotenoids were also affected by the gene expression. AcobGPAT+ and AcobLPAT+ showed a reduced chlorophyll a, chlorophyll b and carotenoid content, suggesting that the carbon flux might have been redirected towards lipid production. Contrarily, the transformant line expressing the three genes simultaneously showed higher chlorophyll and carotenoid content, compared to wild type and the single gene expressing lines. Since the multi-gene expressing line showed a detrimental effect in the photosynthetic efficiency, lower protein and carbohydrate content, we hypothesize that the higher pigment content in the cells may be induced to prevent photoinhibition and oxidative stress.

## Conclusions

In this study, we report the effect of the heterologous overexpression of *LPAT*, *GPAT,* and *DGAT* genes in *N. oleoabundans*. Single and multiple gene expression resulted in an enhanced lipid production for all the transformant lines. We demonstrated that higher neutral lipid content and lipid productivities were achieved when single genes were expressed during nitrogen depleted conditions. Moreover, the single gene expression did not affect growth, biomass productivities and photosynthetic activities significantly. Interestingly, the simultaneous expression of *LPAT*, *GPAT,* and *DGAT* had a negative effect on growth and photosynthetic activity, which resulted in 4-fold bigger cells, lower biomass, and lipid productivities. However, during replete conditions, we observed the highest TAG productivity, which was 4.5-fold higher than wild type. This result indicates that the orchestrated expression of the three genes can induce TAG production during nitrogen replete conditions. However, in order to maximize lipid productivities even further, optimal cultivation conditions and homologous and dual-gene expression need to be investigated in future work. Moreover, future investigations on the alteration of lipid species and precursor pools by the heterologous expression of the Kennedy pathway can help to further elucidate the lipid metabolism in microalgae. Overall, our results provide insights regarding the Kennedy pathway related lipid production and composition, and the improvement of TAG production in microalgae. This approach could lead to the generation of microalgal strains with economical potential for the successful biofuel production.

## Data Availability Statement

All datasets generated for this study are included in the article/supplementary material.

## Author Contributions

CM designed the experiment. CM performed the experiments, analyzed, interpreted the data, and wrote the manuscript. SD’A, RAW, and RHW supervised the project. All authors contributed to the work, discussed the results, read, and approved the final version of this manuscript.

## Funding

This research project was funded by the National Commission of Scientific and Technologic Research of Chile (CONICYT).

## Conflict of Interest

The authors declare that the research was conducted in the absence of any commercial or financial relationships that could be construed as a potential conflict of interest.
